# Tumor regression rate, PD-L1 expression, pembrolizumab/nab-paclitaxel–based regimens, squamous cell carcinoma, and comorbidities were independently associated with efficacy of neoadjuvant chemoimmunotherapy in non-small cell lung cancer

**DOI:** 10.3389/fonc.2022.1057646

**Published:** 2023-01-27

**Authors:** Xingsheng Hu, Chunhong Hu, Xianling Liu, Fang Ma, Junpeng Xie, Ping Zhong, Chenxi Tang, Dan Fan, Yuan Gao, Xiang Feng, Mengge Ding, Dezhi Li, Chaoyuan Liu

**Affiliations:** ^1^ Department of Oncology, The Second Xiangya Hospital of Central South University, Changsha, China; ^2^ Department of Dermatology, North Sichuan Medical College Affiliated Nanchong Central Hospital, Nanchong, China; ^3^ Department of Nursing, North Sichuan Medical College Affiliated Nanchong Central Hospital, Nanchong, China; ^4^ Department of Basic Science, Logan University, Chesterfield, MO, United States; ^5^ Department of Oncology, The Fourth Affiliated Hospital of Zhejiang University School of Medicine, Yiwu, China

**Keywords:** non-small cell lung cancer, neoadjuvant chemoimmunotherapy, surgery, pathologic complete response, major pathologic response

## Abstract

**Background:**

Neoadjuvant chemoimmunotherapy (NCIO) is more effective than neoadjuvant immunotherapy alone for pathological response in non-small cell lung cancer (NSCLC) patients, but the processes for determining patient suitability for its implementation are not clear. We aimed to identify the most relevant factors and build a convenient model to select NSCLC patients who would benefit most from NCIO.

**Methods:**

** **We retrospectively collected the clinical data of patients with locally advanced NSCLC who received NCIO followed by surgery at our institution between January 2019 and July 2022.

**Results:**

A total of 101 eligible stage IIB-IIIC NSCLC patients were included. After NCIO, all patients successfully underwent surgical resection. A total of 46.53% (47/101) of patients achieved pathological complete response (pCR), and 70.30% (71/101) achieved major pathologic response (MPR). Tumor regression rate (adjusted odds ratio OR = 12.33), PD-L1 expression (adjusted odds ratio (OR) = 9.66), pembrolizumab/nab-paclitaxel–based regimens (adjusted OR = 4.92), and comorbidities (adjusted OR = 0.16) were independently associated with pCR rate (all *P* < 0.05). Tumor regression rate (adjusted OR = 8.45), PD-L1 expression (adjusted OR = 5.35), and presence of squamous cell carcinoma (adjusted OR = 7.02) were independently associated with MPR rate (all *P* < 0.05). We established and validated an easy-to-use clinical model to predict pCR (with an area under the curve [AUC] of 0.848) and MPR (with an AUC of 0.847). Of note, the present study showed that CD4^+^ T-cell count/rate and total cholesterol (TC) and high-density lipoprotein cholesterol (HDL-C) levels in the peripheral blood of pre-NCIO patients were also significantly correlated with pathological response in univariate analyses.

**Conclusions:**

The tumor regression rate, PD-L1 expression, pembrolizumab/nab-paclitaxel–based regimens, presence of squamous cell carcinoma, and comorbidities were the main influential factors for incidence of pCR/MPR in patients with stage IIB-IIIC NSCLC in the present study. Through predictive models, we can predict who will benefit most from NCIO prior to the emergence of clinical outcomes in locally advanced NSCLC.

## 1 Introduction

According to 2020 cancer statistics (1), lung cancer was the second most commonly diagnosed cancer and the leading cause of cancer death worldwide. The prognosis of lung cancer is very poor; the 5-year overall survival (OS) rate is only 4–17% (2). Non-small cell lung cancer (NSCLC) accounts for 85% of lung cancers. Most NSCLC patients are diagnosed with locally advanced or metastatic disease, and the 5-year OS rate for locally advanced NSCLC is only 15–25% (3). Loss of radical surgical opportunity is one of the main reasons for poor prognosis. Neoadjauvant chemotherapy can only raise the 5 year OS rate by approximately 5-6% compared to surgery alone. (4). Therefore, exploring new, more effective neoadjuvant treatment regimens is pressing.

The emergence of immunotherapy has already changed the landscape of NSCLC treatment in the past decade. Immune checkpoint inhibitors (ICIs), which are the current leading immunotherapy drugs, have already improved the objective response rate (ORR), progression-free survival (PFS), and OS of advanced NSCLC (5). In 2018, two doses of nivolumab neoadjuvant immunotherapy (NIO) in untreated, resectable stage I-IIIA NSCLC achieved 45% (9/20) major pathologic response (MPR) and 15% (3/20) pathologic complete response (pCR) (6). After this inspiring beginning, the subsequent trial of sintilimab alone also demonstrated similar results (7). In September 2022, a more encouraging result was reported: two doses of neoadjuvant atezolizumab achieved an 80% 3-year OS rate in IB–IIIB NSCLC, although the MPR was only 20% (29/143) (8). Neoadjuvant chemoimmunotherapy (NCIO) may have a better effect, at least in pathological response at present. In the NADIM trial for resectable stage IIIA NSCLC (9), three cycles of neoadjuvant nivolumab plus chemotherapy achieved an MPR of 83% (34/41) and a pCR of 63% (26/41). The PFS rate at 18 or 24 months in the pCR group was significantly higher than that in the non-pCR group (96.2% *vs.* 57.1%, *P* = 0.0023). The OS rate at 18 or 24 months in the pCR/MPR group was also higher than that in the non-pCR group (100% *vs.* 85.7%, *P* = 0.002). In the CheckMate 816 trial, which included IB-IIIA resectable NSCLC (10), compared with neoadjuvant chemotherapy alone group, the NCIO group achieved a higher pCR rate (24.0% *vs.* 2.2%, OR = 13.94, *P* < 0.001), longer median event-free survival (EFS) (31.6 months *vs.* 20. 8 months, HR = 0.63, *P* = 0.005), and OS (both did not reach, HR = 0.57, *P* = 0.008).

Based on these studies, the implementation of NCIO may change the mode of neoadjuvant treatment in NSCLC. However, only a portion of patients can benefit from NCIO, and biomarkers for selecting the most suitable populations of NSCLC are unclear, such as the suitable histology type, stage, PD-L1 expression, and peripheral blood biomarkers. Moreover, previous studies were mainly focused on resectable NSCLC, and studies on locally advanced NSCLC are scarce. Therefore, we carried out this study and hypothesized that efficacy of NCIO would be associated with certain identifiable factors and that these factors might be informative for predicting patients’ benefits prior to the emergence of clinical outcomes in locally advanced NSCLC.

## 2 Materials and methods

### 2.1 Study design and patients

We retrospectively reviewed the clinical records of NSCLC patients who underwent surgery at The Second Xiangya Hospital of Central South University between January 2019 and July 2022. The inclusion criteria were as follows :(1) aged 18 years or older, (2) pathologically confirmed treatment-naive NSCLC, (3) clear clinical stage according to tumor staging (8th edition) of American Joint Committee on Cancer (11) and deemed challenging to resect per multidisciplinary team evaluation, (4) NCIO utilized, (5) postsurgical pathologic response assessment performed, and (6) an Eastern Cooperative Oncology Group score of 0 or 1. Exclusion criteria were as follows: (1) above inclusion criteria not satisfied, (2) presence of distant metastasis (M1 stage), (3) contraindications to immunotherapy, (4) history of other malignant tumors in the past 5 years, and (5) known EGFR/BRAF p.V600E mutations, ALK/ROS1/RET rearrangements, MET amplification, or METex14 skipping mutation. The study adhered to the Declaration of Helsinki and approved by the Review Board and Ethics Commission of The Second Xiangya Hospital of Central South University (2022-K060).

### 2.2 Neoadjuvant chemoimmunotherapy

Patients received the following drugs intravenously: pembrolizumab (200 mg), tislelizumab (200 mg), sintilimab (200 mg), camrelizumab (200 mg), nivolumab (360 mg), toripalimab (240 mg), paclitaxel (135–175 mg/m^2^), paclitaxel liposome (135–175 mg/m^2^), nab-paclitael (260 mg/m^2^), pemetrexed (500 mg/m^2^), gemcitabine (1000 mg/m^2^, d1, d8), carboplatin (AUC, 5; 5 mg/ml per min), cisplatin (80–100mg/m^2^), on day 1 of each 21-day cycle with a total of one to five cycles. Generally, pemetrexed-based chemotherapy/ICI regimens are for adenocarcinoma, and other chemotherapy/ICI regimens are for nonadenocarcinoma.

### 2.3 Outcomes

The primary endpoints were the pCR rate and MPR rate. The definition of pCR was the absence of viable tumor cells in primary and metastatic lymph nodes, and MPR was defined as ≤ 10% of viable tumor cells in the primary tumor bed, regardless of whether there were viable tumor cells in metastatic lymph nodes (12). The radiographic response was assessed according to the Response Evaluation Criteria in Solid Tumors (RECIST v1.1) guidelines (13). PFS was defined as the time from the initiation of NCIO to disease progression or death. OS was defined as the time from the initiation of NCIO to death. AEs were graded according to the NCI-CTCAE (version 4.0).

### 2.4 Statistical analyses

Two groups’ continuous variables with a normal distribution and homogeneity of variance were expressed as the mean ± standard deviation and compared by independent samples *t*-test. Two groups’ continuous variables with a nonnormal distribution or heterogeneity of variance were expressed as the median (interquartile range, IQR) and compared by the Mann−Whitney *U* test. Categorical variables were expressed as numbers (%) and compared by the Chi-square test or Fisher’s exact test. Receiver operating characteristic (ROC) curves were used to assess the predictive power of continuous variables. This study used univariate logistic regression analyses to calculate the odds ratio (OR) value and multivariate logistic analyses to calculate the adjusted OR value. The Kaplan−Meier method was applied to estimate PFS and OS. The above statistical analyses were performed using SPSS 25.0. GraphPad Prism 8 was used to draw and fuse different ROC curves into one figure. MedCalc v20 was used to compare the AUC of the ROC curves. The Rms package of R4.2.1 was used to construct nomograms and calibration curves and perform cross validations, and the pROC package was used to construct the ROC curves and calculate the AUC values of the models. *P* < 0.05 was considered to be statistically significant.

## 3 Results

### 3.1 Patient characteristics

One hundred and one eligible NSCLC patients were included. The clinicopathological characteristics of the patients are presented in **Table 1**.

**Table 1:** Clinicopathological characteristics.

Characteristics	Patients (*n* = 101)
Gender	
Male	93 (92.08%)
Female	8 (7.92%)
Age (years), median (IQR)	58 (55–65)
Smoking history	
No	36 (35.6%)
Yes	65 (64.4%)
Comorbidities	
No	38 (37.62%)
Yes	63 (62.38%)
Differentiation^a^	
Well	9 (9.47%)
Moderate	25 (26.32%)
Moderate–Poor, Poor, undifferentiated	61 (64.21%)
Histology	
Squamous carcinoma	70 (69.3%)
Adenocarcinoma	23 (22.8%)
Adenosquamous	2 (2.0%)
Uncategorized	2 (2.0%)
Others^b^	4 (4.0%)
Clinical T stage	
T1	12 (11.88%)
T2	39 (38.61%)
T3	21 (20.79%)
T3	29 (28.71%)
Clinical N stage	
N0	13 (12.87%)
N1	14 (13.86%)
N2	66 (65.35%)
N3	8 (7.92%)
Clinical TNM stage	
IIB	12 (11.88%)
IIIA	52 (51.49%)
IIIB	33 (32.67%)
IIIC	4 (3.96%)
PD-L1 expression^c^	
<1%	13 (14.29%)
1–49%	48 (52.75%)
≥50%	30 (32.97%)
Median (IQR)	15 (1–60)
Ki-67 expression^d^	
<49%	30 (37.50%)
≥50%	50 (62.50%)
Median (IQR)	60 (40–70)
Treatment cycles	
≤2 cycles	38 (37.62%)
≥3 cycles	63 (62.38%)
Immunotherapy regimens^e^	
Pembrolizumab	37 (37.00%)
Terezumab	30 (30.00%)
Schindelimab	18 (18.00%)
others	15 (15.00%)
Chemotherapy regimens	
Nab-paclitaxel–based	66 (65.35%)
Pemetrexed-based	24 (23.76%)
Others	11 (10.89%)
Radiographic response^f^	
nonORR	17 (18.09%)
ORR	77 (81.91%)
Pathologic response	
pCR	47 (46.53%)
MPR^g^	71 (70.30%)

IQR, interquartile range; PD-L1, programmed death receptor-1 ligand; pCR, pathologic complete response; MPR, major pathologic response; a: six cases were not given exact differentiation grade; b: one large-cell carcinoma, one sarcomatoid carcinoma,1 undifferentiated, one lymphatic epithelioma-like carcinoma; c: 10 cases were not available PD-L1 data; d: 21 cases were not available Ki-67 data; e: one case was not known exact name of PD1 inhibitor; f: seven cases cannot be exact assessed; g: MPR included 50 pCR cases.

### 3.2 Neoadjuvant chemoimmunotherapy and adverse events

Patients received a median of three cycles of NCIO before surgery. Among them, two patients only received one cycle of NCIO due to an allergic reaction after treatment, but they continued with treatment and completed two and three cycles of chemotherapy, respectively. Thirty-six and 37 patients received two and three cycles of NCIO, respectively. Twenty and six patients received four and five cycles of NCIO, respectively. During NCIO, the most common grades 1–2 AEs were anemia (66.34%), hyperbilirubinemia (63.37%), and alopecia (46.53%). The most common grade 3 or worse AEs were anorexia (7.92%), peripheral sensory neuropathy (4.95%), and nausea/vomiting (3.96%) (**Table 2**). No deaths were observed.

**Table 2:** Adverse events during neoadjuvant immunochemotherapy.

Variables	Grades 1–2		Grades 3–4	
Symptoms				
Nausea	38 (37.62%)		4 (3.96%)	
Vomiting	24 (23.76%)		4 (3.96%)	
Anorexia	33 (32.67%)		8 (7.92%)	
Diarrhea	13 (12.87%)		2 (1.98%)	
Constipation	23 (22.77%)		3 (2.97%)	
Fatigue	27 (26.73%)		2 (1.98%)	
Alopecia	47(46.53%)		0 (0.00%)	
Pruritus	35 (34.65%)		3 (2.97%)	
Rush	21 (20.79%)		2 (1.98%)	
Arthralgia or myalgia	21 (20.79%)		1 (0.99%)	
Peripheral sensory neuropathy	34 (33.66%)		5 (4.95%)	
Laboratory examinations				
Aleucocytosis	16 (15.84%)		4 (3.96%)	
Neutropenia	13 (12.87%)		4 (3.96%)	
Febrile neutropenia	1 (0.99%)		0 (0.00%)	
Anemia	67 (66.34%)		2 (1.98%)	
Thrombocytopenia	26 (25.74%)		3 (2.97%)	
Increased ALT	26 (25.74%)		1 (0.99%)	
Increased AST	21 (20.79%)		2 (1.98%)	
Increased total bilirubin	9 (8.91%)		1 (0.99%)	
Hyperbilirubinemia	64(63.37%)		0 (0.00%)	
Increased creatinine	2 (1.98%)		0 (0.00%)	
Hyponatremia	20 (19.80%)		1 (0.99%)	
Kaliopenia	11 (10.89%)		1 (0.99%)	
Hypocalcemia	28 (27.72%)		0 (0.00%)	
Hyperglycemia	6 (5.94%)		0 (0.00%)	
Hypophosphatemia	14 (13.86%)		0 (0.00%)	
Hypothyroidism	8 (7.92%)		0 (0.00%)	
Adrenal insufficiency	11 (10.89%)		0 (0.00%)	

ALT, Alanine aminotransferase; AST, Aspartate aminotransferase.

### 3.3 Surgical outcomes and complications

After NCIO, all patients underwent surgical resection; the R0 resection rate was 100% (101/101), and the N2 patients’ downstaging rate was 86.57% (58/67). The most common perioperative complications were pneumothorax (21.78%), pleural effusion (17.82%), and chylothorax (3.96%), with no deaths at 30 or 90 days after surgery (**Complementary Table 1**).

### 3.4 Radiographic and pathological response assessments

After NCIO, seven patients were excluded from radiographic response assessment due to unavailability of precise tumor size data, since tumor lesions combined atelectasis, patches of inflammation shadows or radiographic data were kept in other hospitals.

The complete response (CR), partial response (PR), stable disease (SD), and progressive disease (PD) rates were 7.45% (7/94), 74.47% (70/94), 18.09% (17/94), and 0.00% (0/94), respectively. The ORR rate was 81.94% (77/94), and the disease control rate (DCR) was 100% (94/94). After surgery, all patients’ specimens were subjected to pathological response assessment. The pCR rate was 46.53% (47/101), and the MPR rate was 70.30% (71/101) (including 47 pCR patients).

### 3.5 Recurrence and survival

**Figure 1:**
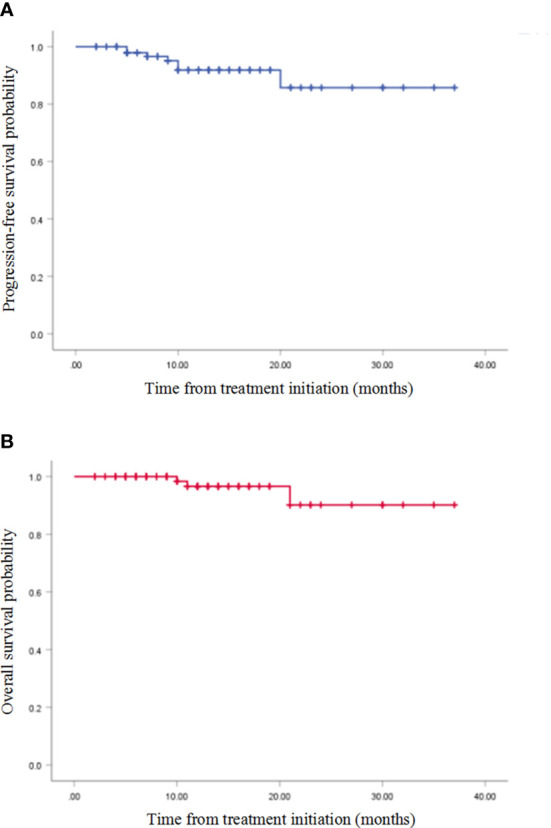
Kaplan–Meier survival curves. **(A)** Disease-free survival; **(B)** overall survival.

Until September 2022, the median follow-up time was 12 (IQR: 7–16) months. Seven patients relapsed, and three patients died due to disease progression. The median PFS and OS were not reached, and the 1-year PFS and 1-year OS rates were 91.80% and 96.60%, respectively (**Figure 1**).

### 3.6 Factors associated with pCR or MPR

The associations between clinicopathological factors and the pCR and MPR are presented in **Table 3**. Many factors were significantly associated with the pCR and MPR. Briefly, histology type, PD-L1 expression of pre-NCIO, pembrolizumab/nab-paclitaxel–based regimens, tumor size of post-NICO, tumor regression rate, ORR, max of standardized uptake value (SUVmax) of tumor post-NCIO, △SUVmax rate (SUVmax reduced rate) of tumor post-NICO were commonly associated with the pCR and MPR rates (all *P* < 0.05). In addition, comorbidities, differentiation grade, hemoglobin, and CD4^+^ T-cell count/rate of pre-NCIO were specifically significantly associated with the pCR rate (all *P* < 0.05); carcinoembryonic antigen (CEA), total cholesterol (TC), and high-density lipoprotein cholesterol (HDL-C) of pre-NCIO were specifically significantly associated with the MPR rate (all *P* < 0.05).

**Table 3:** Clinicopathological factors were associated with pCR or MPR.

Variables	pCR (*n* = 47)	non-PCR (*n* = 54)	*P*	MPR (*n* = 71)	non-MPR (*n* = 30)	*P*
Sex						
Male	43 (46.24%)	50 (53.76%)		66 (70.97%)	27 (29.03%)	
Female	4 (50.00%)	4 (50.00%)	1.000	5 (62.50%)	3 (37.50%)	0.921
Age (years)	61 (56–66)	58 (54–64)	0.192	58 (55–66)	58 (55–62)	0.517
Smoking history						
No	16 (44.44%)	20 (55.56%)		22 (61.11%)	14 (38.89%)	
Yes	31 (47.69%)	34 (52.31%)	0.754	49 (75.38%)	16 (24.62%)	0.133
Comorbidities						
No	24 (63.16%)	14 (36.84%)		28 (73.68%)	10 (26.32%)	
Yes	23 (36.51%)	40 (63.49%)	0.009	43 (68.25%)	20 (31.75%)	0.563
Differentiation grade^a^						
Well/moderate	11 (32.35%)	23 (67.65%)		20 (58.82%)	14 (42.18%)	
Moderate–poor/ poor/undifferentiated	36 (53.73%)	31 (46.27%)	0.042	51 (76.12%)	16 (23.88%)	0.072
Histology type^b^						
Squamous cell carcinoma	39 (55.71%)	31 (44.29%)		56 (80.00%)	14 (20.00%)	
Nonsquamous cell carcinoma	7 (25.93%)	20 (64.07%)	0.008	12 (44.44%)	15 (55.56%)	0.001
Clinical T stage						
T1+T2	25 (49.02%)	26 (50.08%)		35 (68.62%)	16 (31.38%)	
T3+T4	22 (44.00%)	28 (66.00%)	0.613	36 (72.00%)	14 (28.00%)	0.711
Clinical N stage						
N0	5 (38.46%)	8 (61.54%)		6 (46.15%)	7 (53.85%)	
N1-3	42 (47.73%)	46 (52.27%)	0.532	65 (73.86%)	23 (26.14%)	0.086
Clinical TNM stage						
IIB-IIIA	30 (46.88%)	34 (53.12%)		41 (64.06%)	23 (35.94%)	
IIIB-IIIC	17 (45.95%)	20 (54.05%)	0.928	30 (81.08%)	7 (18.92%)	0.071
PD-L1 expression (%)	40.00 (5.00–76.25)	5.00 (1.0–57.50)	0.013	30.0 (2.0–70.0)	5 (0.75–35.0)	0.040
Ki-67 expression (%)	60.0 (42.5–70.0)	50.00 (35.0–70.0)	0.115	60.0 (40.0–70.0)	40.00 (31.2–70.0)	0.076
WBC count (*10^9^/l)	6.90 (5.90–7.90)	7.50 (5.80–8.50)	0.721	7.00 (5.90–8.40)	6.90 (5.30–8.20)	0.323
Hemoglobin (g/l)	137.50 ± 14.60	143.70 ± 15.70	0.042	139.10 ± 14.90	144.90 ± 16.40	0.083
Lymphocyte count (*10^9^/l)	1.65 ± 0.46	1.72 ± 0.50	0.472	1.67 ± 0.45	1.73 ± 0.55	0.610
CEA (ng/ml)	2.46 (1.48–4.00)	3.17 (2.11–5.88)	0.053	2.62 (1.53–4.08)	3.60 (2.58–8.68)	0.042
CYFRA21-1 (ng/ml)	3.71 (1.40–5.28)	3.26 (1.36–6.48)	0.862	3.71 (1.95–6.05)	2.20 (1.06–6.04)	0.288
SCC-Ag (ng/ml)	1.47 (0.82–2.82)	1.27 (0.70–2.28)	0.638	1.43 (0.83–2.53)	1.20 (0.67–2.04)	0.421
Interleukin-6 (pg/ml)	6.33 (3.98–12.20)	7.83 (3.40–11.73)	0.969	6.85 (4.13–12.70)	5.75 (1.93–10.60)	0.520
CRP (mg/l)	5.96 (3.22–17.90)	6.45 (2.70–15.02)	0.898	8.75 (3.38–17.60)	4.24 (2.11–9.54)	0.147
Triglycerides (mmol/l)	1.56 ± 0.76	1.77 ± 0.80	0.330	1.47 (0.98–2.35)	1.71 (1.25–2.18)	0.515
Total cholesterol (mmol/l)	4.88 ± 0.84	4.75 ± 1.08	0.636	4.62 ± 0.95	5.23 ± 0.92	0.027
HDL-C (mmol/l)	1.13 ± 0.24	1.12 ± 0.28	0.975	1.08 ± 0.26	1.23 ± 0.25	0.041
LDL-C (mmol/l)	3.28 ± 0.82	3.12 ± 0.92	0.488	3.04 ± 0.91	3.52 ± 0.70	0.054
HDL-C/Total cholesterol (%)	0.22 (0.19–0.28)	0.23 (0.21–0.26)	0.714	0.22 (0.19–0.28)	0.24 (0.21–0.26)	0.593
T-cell count (*10^3^/µl)	1.13 ± 3.87	1.34 ± 4.57	0.122	1.19 ± 4.23	1.37 ± 4.46	0.247
T-cell rate (%)	66.65 ± 9.51	70.20 ± 7.08	0.196	67.64 ± 8.74	70.53 ± 7.78	0.362
CD4^+^ T-cell count (*10^2^/µl)	6.19 ± 2.26	8.32 ± 3.07	0.018	6.74 ± 2.53	8.64 ± 3.41	0.071
CD4^+^ T-cell rate (%)	37.30 ± 8.50	43.50 ± 8.30	0.025	39.10 ± 8.96	43.70 ± 8.20	0.158
CD8^+^ T-cell count (*10^2^/µl)	4.02(3.09–5.52)	4.84 (3.17–5.92)	0.687	3.97 (2.98–5.75)	5.02(3.51–5.94)	0.418
CD8^+^ T-cell rate (%)	27.00 (18.50–35.00)	25.00 (20.00–30.00)	0.810	25.50 (18.75–29.75)	26.00 (20.00–31.00)	0.794
CD4^+^/CD8^+^ T-cell ratio(%)	1.57 ± 0.69	1.88 ± 0.70	0.175	1.69 ± 0.73	1.81 ± 0.65	0.633
B cell count (*10^2^/µl)	1.48 (1.02–2.43)	1.68 (1.04–2.61)	0.667	1.51 (1.17–2.53)	1.55 (0.95–2.29)	0.887
B cell rate (%)	10.12 ± 3.50	9.50 ± 4.54	0.834	10.00 (7.75–13.11)	7.00 (5.50–14.25)	0.414
NK cell count (*10^2^/µl)	3.21(2.47–3.86)	2.89 (1.82–4.10)	0.708	3.12 (2.33–4.00)	2.85 (1.80–4.37)	0.764
NK cell rate (%)	20.00 (15.00–24.00)	15.00 (13.00–22.00)	0.270	20.0 (14.0–24.0)	14.75 (12.75–23.23)	0.363
NCIO regimens						
Pembrolizumab/nab- paclitaxel–based	26 (36.62%)	45 (63.38%)		45 (63.38%)	26 (36.62%)	
Others^c^	21 (72.41%)	8 (27.59%)	0.001	26 (89.66%)	3 (10.34%)	0.009
Treatment cylce						
≤2 cycles	14 (36.84%)	24 (63.16%)		23 (60.53%)	15 (39.47%)	
>2 cycles	33 (52.38%)	30 (47.62%)	0.129	48 (76.19%)	15 (23.81%)	0.095
≤3 cycles	33 (44.00%)	42 (66.00%)		51 (68.00%)	24 (32.00%)	
>3 cycles	14 (53.85%)	12 (46.15%)	0.386	20 (76.92%)	6 (23.08%)	0.391
Tumor size of pre-NCIO (mm)	61.95 (39.00–74.68)	50.00 (39.00–64.00)	0.310	60.00 (39.00–82.00)	49.90 (38.75–58.98)	0.143
Tumor size of post-NICO (mm)	25.0 (15.00–35.00)	31.00 (24.00–42.00)	0.010	27.75 (16.75–36.00)	31.50 (24.48–41.75)	0.035
Tumor regression rate (%)	58.2 ± 19.6	37.9 ± 23.5	<0.001	55.0 ± 20.3	29.0 ± 22.4	<0.001
Radiographic response^d^						
nonORR	1 (5.90%)	16 (94.10%)		4 (23.53%)	13 (76.47%)	
ORR	42 (54.55%)	35 (45.45%)	<0.001	62 (80.52%)	15 (19.48%)	<0.001
SUVmax of tumor pre-NCIO	22.53 ± 10.37	22.11 ± 10.40	0.879	22.40 ± 9.16	22.10 ± 12.49	0.918
SUVmax of tumor post-NCIO	3.20 (1.35–5.90)	9.11 (5.18–14.98)	<0.001	4.55 (1.95–7.43)	15.20 (7.91–23.60)	<0.001
△SUVmax rate of tumor post-NCIO (%)	82.96 (77.54–1.00)	56.80 (44.61–79.88)	0.004	80.21 (77.35–1.00)	53.22 (-4.36–66.47)	<0.001

a: Six cases were not given exact differentiation grade; b: two uncategorized and two adenosquamous cases were not entered into the calculation; c: one case was not known exact name of PD1 inhibitor, did not enter computer; d: seven cases cannot be exact assessed; PD-L1: programmed death receptor-1 ligand; WBC, white blood cell; CEA ,carcinoembryonic antigen; CYFRA21-1, cytokeratin fragment antiogen 21-1; SCC-Ag, squamous cell carcinoma-associated antigen; CRP, C-reactive protein; HDL-C, high-density lipoprotein cholesterol; LDL-C, low-density lipoprotein cholesterol; NCIO, neoadjuvant chemoimmunotherapy; ORR, objective response rate; SUV, standardized uptake value; △SUVmax = SUVmax of pre–NCIO-SUVmax of post-NCIO.

We also found that pembrolizumab/chemotherapy regimens were more effective than other ICI/chemotherapy regimens, and nab-paclitaxel–based/ICI regimens were more effective than other chemotherapy/ICI regimens (**Complementary Table 2**).

### 3.7 ROC curve analyses

**Figure 2:**
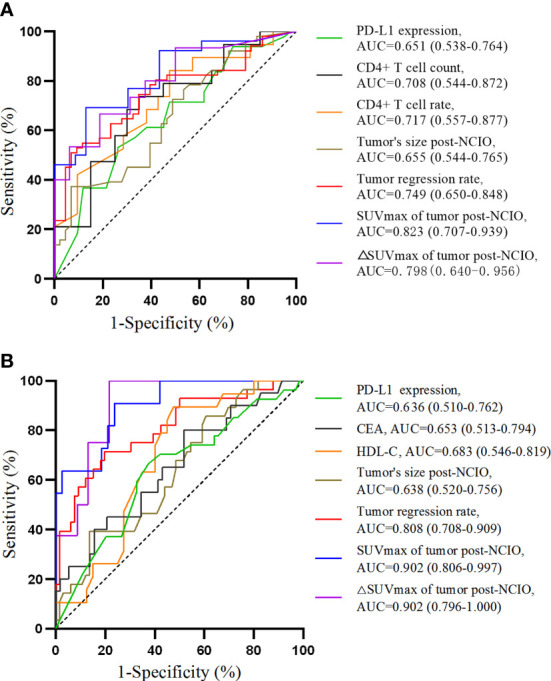
Receiver operating characteristic curves of univariate analyses. **(A)** For pathological complete response; **(B)** for major pathologic response. AUC, area under curve.

Regarding the above significant continuous variables, we further analyzed their predictive ability and cutoff values for pCR/MPR with ROC curves. The results showed that except for hemoglobin level (*P* = 0.080) and TC level (*P* = 0.078) of pre-NCIO, all of the other continuous variables had significant predictive ability (all *P* < 0.05) (**Table 4** and **Figure 2**). In addition, we compared the AUCs of the ROC curves. Briefly, for both pCR and MPR, the AUC of SUVmax of tumor post-NCIO was significantly larger than that of PD-L1 expression of pre-NCIO and tumor size of post-NCIO (all *P* < 0.05); and the AUC of △SUVmax rate of tumor post-NICO was significantly larger than that of PD-L1 expression of pre-NCIO (*P* < 0.05). In addition, for MPR alone, the AUC of tumor regression rate, SUVmax of tumor post-NCIO, and △SUVmax rate of tumor post-NCIO were significantly larger than the CEA of pre-NCIO, HDL-C of pre-NCIO, and tumor size of post-NCIO (all *P* < 0.05) (**Complementary Table 3**). After dividing cutoff values, we compared continuous variables again by the Chi-square test, and all of them demonstrated statistical significance (**Complementary Table 4**).

**Table 4:** Receiver operating characteristic curves analyses for pCR or MPR.

Variables	Cutoff value	Sensitivity	Specificity	*P*	Cutoff value	Sensitivity	Specificity	*P*
PD-L1 expression (%)	9.00	0.74	0.53	0.013	12.50	0.63	0.67	0.041
CD4^+^ T-cell count (*10^2^/µl)	6.75	0.65	0.74	0.026	–	–	–	–
CD4^+^ T-cell rate (%)	36.50	0.52	0.84	0.019	–	–	–	–
CEA (ng/ml)	–	–	–	–	2.54	0.48	0.80	0.042
HDL-C (mmol/l)	–	–	–	–	1.04	0.53	0.90	0.024
Tumor size of post-NCIO (mm)	37.50	0.93	0.37	0.010	0.40	0.86	0.39	0.035
Tumor regression rate (%)	36.89	0.93	0.51	<0.001	39.74	0.81	0.71	<0.001
	30.45	0.95	0.33	<0.001	30.45	0.92	0.50	<0.001
SUVmax of tumor post-NCIO	6.75	0.87	0.69	<0.001	7.43	0.76	0.91	<0.001
△SUVmax rate of tumor post-NCIO (%)	76.76	0.81	0.67	0.005	76.76	0.78	1.00	0.001

PD-L1: programmed death receptor-1 ligand; CEA, carcinoembryonic antigen; HDL-C, high-density lipoprotein cholesterol; NCIO, neoadjuvant chemoimmunotherapy; SUV, standardized uptake value; △SUVmax = SUVmax of pre–NCIO-SUVmax of post-NCIO.

We also compared the pCR and MPR rate at PD-L1 expression levels of 1%, 25%, and 50%, and the *P*-value was only significant for MPR when PD-L1 expression was 25% (*P* = 0.040) (**Complementary Table 5**), and its corresponding OR value was 2.69 (*P* = 0.043).

### 3.8 Univariate and multivariate logistic regression analyses

Regarding the above significant continuous variables in ROC curves and categorical variables associated with pCR or MPR, we further tested OR values in univariate logistic analyses. As a result, only the differentiation grade was not significant (*P* = 0.075), and all the other variables were significant (all *P* < 0.05) (**Table 5**). In particular, the OR of △SUVmax rate of tumor post-NCIO could not be exactly calculated due to 100% specificity at the cutoff value (76.76%), and no positive cases were present in the non-MPR group, so we excluded it from further multivariate analyses.

**Table 5:** Univariate logistic regression of factors was associated with pCR or MPR.

Variables	pCR *vs.* non-pCR		Variables	MPR vs. non-MPR	
	OR (95% CI)	*P*		OR (95% CI)	*P*
Comorbidities (Yes *vs.* No)	0.34 (0.15–0.77)	0.010	–	–	–
Differentiation grade (Poor *vs.* Well/Moderate)	–	0.075	–	–	–
Squamous cell carcinoma (Yes *vs.* No)	3.59 (1.35–9.59)	0.011	Squamous cell carcinoma (Yes *vs.* No)	5.00 (1.92–13.04)	0.001
PD-L1 expression (%) (≥9 *vs.* <9)	3.19 (1.31–7.74)	0.011	PD-L1 expression (%) (≥12.5 *vs.* <12.5)	3.33 (1.29–8.59)	0.013
CD4^+^ T-cell count (*10^2^/µl) (<6.75 *vs.* ≥6.75)	5.20 (1.32–20.54)	0.019	CEA (ng/ml) (<2.54 *vs.* ≥2.54)	4.00 (1.20–13.37)	0.024
CD4^+^ T-cell rate (%) (<36.50 *vs.* ≥36.50)	5.87 (1.31–26.33)	0.021	HDL-C (mmol/l) (<1.04 *vs.* ≥1.04)	9.40 (1.91–46.12)	0.006
Pembrolizumab/nab-paclitaxel–based regimen (Yes *vs.* No)	4.05 (1.56–10.49)	0.004	Pembrolizumab/nab-paclitaxel–based regimen (Yes *vs.* No)	4.70 (1.29–17.07)	0.019
Tumor size of post-NCIO (mm) (<37.5 *vs.* ≥37.5)	7.92 (2.15–29.15)	0.002	Tumor size of post-NCIO (mm) (<40.4 *vs.* ≥40.4)	4.10 (1.46–11.53)	0.008
Tumor regression rate (%) (≥36.89 *vs.* <36.89)	13.87 (7.80–50.65)	<0.001	Tumor regression rate (%) (≥39.74 *vs.* <39.74)	10.19 (3.68–28.26)	<0.001
SUVmax of tumor post-NCIO (<6.75 *vs.* ≥6.75)	15.75 (3.63–68.41)	<0.001	SUVmax of tumor post-NCIO (<7.43 *vs.* ≥7.43)	33.33 (3.74–296.78)	0.002
△SUVmax rate of tumor post-NCIO (%) (76.76≥ *vs.* <76.76)	8.67 (1.66–45.21)	0.010	△SUVmax rate of tumor post-NCIO (%) (76.76≥ *vs.* <76.76)	–	–

PD-L1: programmed death receptor-1 ligand; CEA, carcinoembryonic antigen; HDL-C, high-density lipoprotein cholesterol; ICIs, immune checkpoint inhibitors; SUV, standardized uptake value; △SUVmax = SUVmax of pre–NCIO-SUVmax of post-NCIO.

In addition, it was essential to have a suitable number of factors for entry into multivariate analyses. Therefore, we further excluded a relative small number of factors, that is, CD4^+^ T-cell count of pre-NCIO (*n* = 39), CD4^+^ T-cell rate of pre-NCIO (*n* = 40), SUVmax of tumor post-NCIO (*n* = 50) and △SUVmax rate of tumor post-NCIO (*n* = 31), even though they had significance in univariate analyses. Then, the remaining six significant factors (comorbidities, presence of squamous cell carcinoma (SCC), PD-L1 expression of pre-NCIO, pembrolizumab/nab-paclitaxel–based regimens, tumor size of post-NCIO, and tumor regression rate) of pCR and seven significant factors (presence of SCC, PD-L1 expression of pre-NCIO, CEA of pre-NCIO, HDL-C of pre-NCIO, pembrolizumab/nab-paclitaxel–based regimens, tumor size of post-NCIO, tumor regression rate) of MPR were chosen for the multivariate logistic regression model. Finally, the results showed that comorbidities (adjusted OR = 0.16), PD-L1 expression of pre-NCIO (adjusted OR = 9.66), pembrolizumab/nab-paclitaxel–based regimens (adjusted OR = 4.92), and tumor regression rate (adjusted OR = 12.33) were independently associated with pCR (all *P* < 0.05). Presence of SCC (adjusted OR = 7.02), PD-L1 expression of pre-NCIO (adjusted OR = 5.35), and tumor regression rate (adjusted OR = 8.45) were independently associated with MPR (all *P* < 0.05) (**Table 6**).

**Table 6:** Multivariate logistic regressions of factors were associated with pCR or MPR.

Variables	pCR *vs.* non-pCR		MPR *vs.* non-MPR	
	OR (95% CI)		P	OR (95% CI)	P
Comorbidities (Yes *vs.* No)	0.16 (0.40–0.60)	0.007	–	–
Squamous cell carcinoma (Yes *vs.* No)	–	–	7.02 (1.82–27.12)	0.005
PD-L1 expression (%) (≥9.0 *vs.* <9.0) ^a^ (≥12.5 *vs.* <12.5)^b^	9.66 (2.26–41.24)	0.002	5.35 (1.44–19.86)	0.012
Pembrolizumab/nab-paclitaxel–based regimen (Yes *vs.* No)	4.92 (1.19–20.26)	0.028	–	–
Tumor regression rate (%) (≥36.89 *vs.* <36.89)^a^ (≥39.74 *vs.* <39.74)^b^	12.33 (2.82–53.87)	0.001	8.45 (2.34–30.52)	0.001

a: For pCR vs. nonpCR; b: for MPR vs. nonMPR; PD-L1: programmed death receptor-1 ligand.

### 3.9 Clinical predictive models

**Figure 3:**
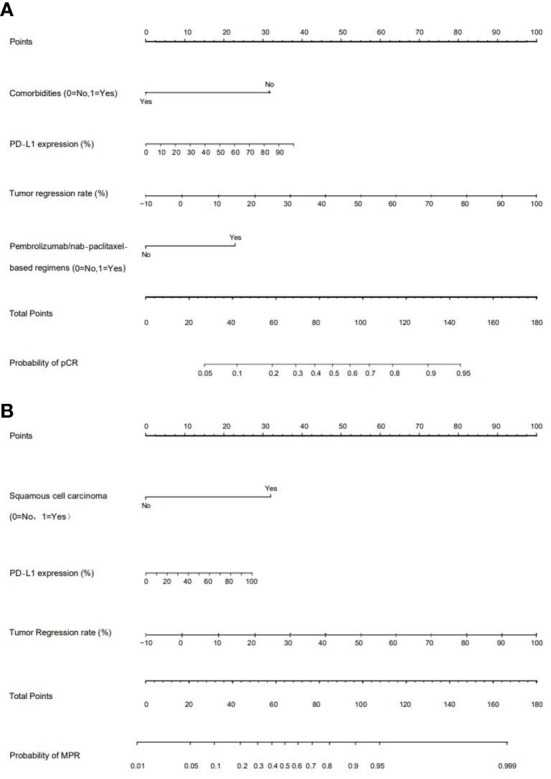
Nomograms of predictive models. **(A)** For pathological complete response (pCR) model; **(B)** for major pathologic response (MPR) model. PD-L1: programmed death receptor-1 ligand.

**Figure 4:**
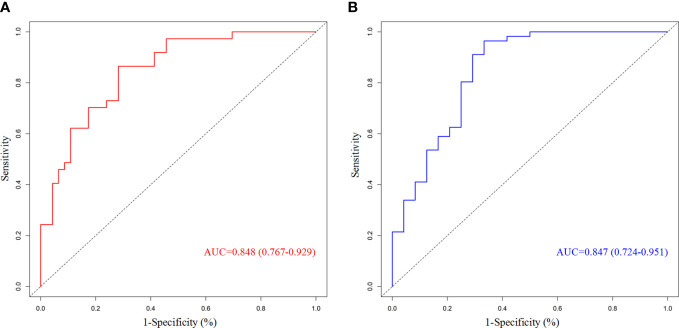
Receiver operating characteristic curves of predictive model. **(A)** For pathological complete response; **(B)** for major pathologic response. AUC, area under curve.

**Figure 5:**
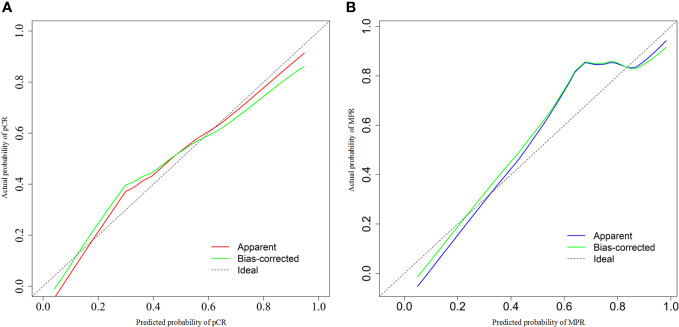
Calibration curves of predictive model. **(A)** For pathological complete response model; **(B)** for major pathologic response model. AUC, area under curve.

We chose significant independent factors to establish clinical predictive models. In the nomogram, first, we determined each factor’s corresponding points, then summed them to obtain the total points and finally determined the total points corresponding to the probability of pCR/MPR (**Figure 3**). The AUC of the pCR model was 0.848, and the AUC for the MPR model was 0.847, both satisfactory (**Figure 4**). The calibration curves showed that the apparent/bias-corrected curves basically approached ideal curves (**Figure 5**). We carried out 1000 times repetitions 1/10 cross-validations, and the mean AUCs of pCR and MPR were 0.789 and 0.809, respectively. The total accuracies were 0.728 and 0.829, respectively, which indicated that the models were satisfactory and steady.

### 3.10 Exploratory analyses

**Figure 6:**
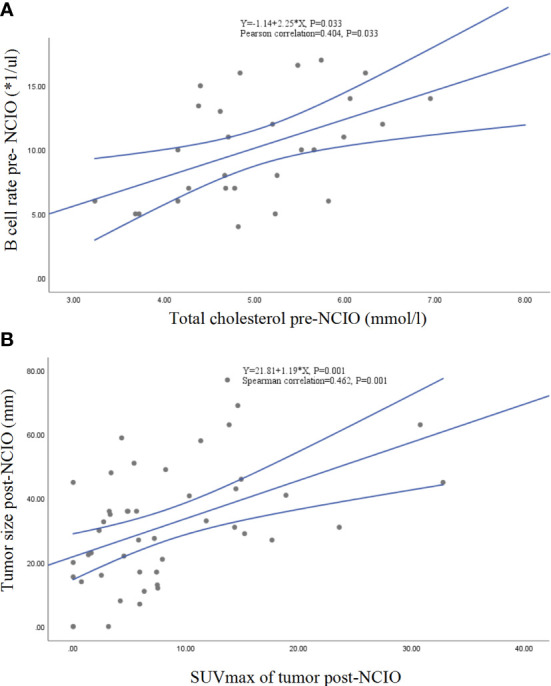
Linear correlations and regressions. **(A)** Total cholesterol and B-cell rate of pre-NCIO; **(B)** SUVmax and tumor size of post-NCIO. NCIO: neoadjuvant chemoimmunotherapy, SUV, standardized uptake value.

In an exploratory analysis, we found that the SUVmax of tumor post-NCIO was significantly associated with tumor size of post-NCIO (Spearman correlation = 0.462, *P* = 0.001), TC of pre-NCIO was significantly associated with B-cell count of pre-NCIO (Pearson correlation = 0.404, *P* = 0.033) and NK-cell count of pre-NCIO (Spearman correlation = -0.396, *P* = 0.041), HDL-C of pre-NCIO had a trend of correlation with CD8^+^ T-cell rate of pre-NCIO (Spearman correlation = 0.3142, *P* = 0.070), CEA of pre-NCIO was significantly associated with T count of pre-NCIO (Spearman correlation = -0. 375, *P* = 0.045) and NK count of pre-NCIO (Spearman correlation = -0.400, *P* = 0.032), comorbidities group had a trend of higher B cell count of post-NCIO (median 127.50/µl *vs.* 92.00/µl, *P* = 0.072). Finally, the linear regression equations of two pairs of significant dependent factors (SUVmax of tumor post-NCIO and tumor size of post-NCIO, TC of pre-NCIO and B-cell count of pre-NCIO) were established (all *P* < 0.05) (**Figure 6**). Other pairs of dependent factors had trends of significance in linear regression equations (data not shown).

## 4 Discussion

Compared with adjuvant treatment, NIO has an advantage in increasing the release or exposure of cancer neoantigens to activate an adaptive antitumor response (14). Meanwhile, real-time assessment of treatment effects may provide a useful reference for adjuvant treatment. Currently, NIO alone can achieve an MPR rate of 20–45% and a pCR rate of 6–16% (6–8, 15), and NICO can achieve an MPR rate of 40–83% and a pCR rate of 18–63% (9, 16–19). NIO and NCIO had similar surgical resection rates (83–100%), R0 rates (87–100%) and grade 3 or higher AE rates (0–33%) (6–9, 15–19). Generally, the MPR or pCR rate of NCIO was higher than that of NIO alone. The survival data of most of the studies were not mature; some studies reported ≤ 2 years of data, and one study reported 3-year data. The 18-month PFS rate was 73% in the Checkmate 159 study (6), and the 3-year OS rate was 80% in the newest report of atezolizumab monotherapy (8). The 2-year PFS/EFS rate was 45.8–84.4%, and the 2-year OS rate was 79.9–94.1% in NCIO studies (9, 17, 18, 20). MPR/pCR was proven to be meaningful in terms of survival benefit in some studies (9, 10, 20).

In our study, NCIO achieved a 70.30% MPR, 46.53% pCR, 81.94% ORR, 100% DCR, and 100% surgery/R0 resection rate; AEs were manageable. Many factors were found to be significantly associated with the pCR/MPR rate, five of which were independent factors. Of note, among these factors, we first reported several new indicators: pembrolizumab/nab-paclitaxel–based regimens, comorbidities, peripheral blood CD4^+^ T-cell count/rate, TC, HDL-C and CEA before treatment. In addition, we established predictive models for pCR and MPR with AUCs of 0.848 and 0.847, respectively. We found that the predictive accuracy of the MPR model (0.829) was higher than that of the pCR model (0.728), which may be due to inclusion of some non-pCR patients with a 0–10% pathological response.

The tumor regression rate, the most influential factor in the present study, it independently associated with both pCR and MPR, and it accounted for the largest weight in the nomogram predictive model. When its cutoff value was 36.89% for pCR and 39.74% for MPR, the incidence of pCR and MPR were alone increased 12.33 and 8.45 times. Compared with the ORR criterion (≥ 30% regression), the optimal cutoff value of the tumor regression rate may more easily reflect the correlation between radiographic response and pathological response. In our study, when the cutoff value of the tumor regression rate was 36.89% for pCR, the specificity of predictive ability (0.51) was higher than that of the ORR criterion (0.33), but sensitivity values were similar (0.93 and 0.95). When the cutoff was 39.74% for MPR, the specificity of predictive ability (0.71) was also higher than that of the ORR criterion (0.50), but their sensitivity values were mildly different (0.80 and 0.92). In addition, the AUC of the tumor regression rate was significantly larger than that of PD-L1 expression, CEA, HDL-C of pre-NCIO, and tumor size of post-NCIO. The NCT02716038 (16), SAKK 16/14 (17) and NEOSTAR (21) trials also showed a positive correlation between the tumor regression rate and pathological response.

PD-L1 expression of pre-NCIO, the second most influential factor, was also independently associated with both pCR and MPR in our study. When its cutoff value was 9% for pCR and 12.5% for MPR, the incidences of pCR and MPR were alone increased 9.66 times and 5.35 times, respectively, in multivariate analyses. These results indicated that PD-L1 expression may be more critical for assessing the complete removal of tumors. When PD-L1 expression was 1, 25, and 50%, the *P*-value was only significant for the MPR rate comparison at 25%, but its corresponding OR value (2.69) was lower than its OR value (3.64) at the optimal cutoff value of 12.5%. Therefore, our suggestion is that an optimal cutoff value of PD-L1 expression may also be better than conventional categorical boundaries. Concordantly, the positive association of PD-L1 expression and pathological response was also proven in the NADIM (9), CheckMate 816 (10), SAKK 16/14 (17), and NEOSTAR trials (21).

The pembrolizumab/nab-paclitaxel–based regimens was significantly associated with pCR/MPR and was an independent factor for pCR. To our knowledge, no other study of NCIO compared these regimens with other ICI/chemotherapy regimens. However, our finding was consistent with studies of metastatic NSCLC. Some network meta-analyses have proven that the pembrolizum-ab/chemotherapy regimen has the highest probability of offering the best OS (probability of 65–98.5%), PFS (probability of 47–69%), and ORR (probability of 65.0%) among different ICI/chemotherapy or ICI-alone regimens when compared with chemotherapy alone (22–24). A phase III trial that studied stage IIIB-IV NSCLC stated that nab-paclitaxel chemotherapy regimen had a significantly higher ORR than solvent-based paclitaxel in all NSCLC patients (33% vs. 25%; P = 0.005) and SCC patients (41% *vs.* 24%; *P* < 0.001). The OS of nab-paclitaxel was longer in the North American subgroup (12.7 *vs.* 9.8 months, *P* = 0.008) (25).

The presence of SCC was associated with pCR/MPR in univariate analyses and was an independent factor affecting MPR in multivariate analyses, alone increasing the MPR rate 7.02 times. In Wu’s study (19), more SCC cases were observed in the MPR group (*P* = 0.008). The MPR rate with SCC had an obviously higher trend than with adenocarcinoma in the NCT02716038 trial (16) (80% *vs.* 53%, *P* = 0.17) and Gao’s study (48.4% *vs.* 0%, *P* = 0.067) (7). To reduce the influence of actionable driver oncogene mutations, we excluded patients with actionable driver oncogene mutations from our study. We found that patients with known actionable driver oncogene mutations experienced poor efficacy in NCIO; all of three patients with RET rearrangements and one patient with ROS1 rearrangements were non-MPR, only one patient with BRAF mutations achieved pCR (**Complementary Table 6**). However, KRAS mutation did not influence the efficacy, and the MPR rate was 62.5% (three cases were pCR) in eight patients with KRAS mutation in the present study.

Interestingly, compared with the non-pCR group, we found that patients in the pCR group had a significantly lower circulating^CD4+^ CD4^+^ T-cell rate/count before NCIO. Furthermore, CD4^+^ T-cell count/rate had a significant predictive ability for pCR (AUC = 0.733 and 0.732). Meanwhile, we found that other immune cell (T-cell count/rate, CD8^+^ T-cell and B-cell count) before NCIO also demonstrated a lower trend in the pCR and MPR group than in the reciprocal group. However, the NK-cell count/rate before NCIO presented an inverse trend. A higher circulating NK-cell count before NCIO was associated with a better pathological response (26). However, why did the pCR/MPR group have few CD4^+^ or CD8^+^ T cells? Was it just a coincidence, or were there some significant correlations? Some relevant reports indicated that this circumstance did indeed correlate with better outcomes, possibly due to the homing of circulating immune cells to the tumor microenvironment (TME) after immunotherapy. A study of stage IV melanoma patients receiving ICI treatment showed that early disappearance of tumor-associated antigen reactive T- cells (CD4^+^ T-cells, CD8^+^-T cells, *etc.*) from peripheral blood was associated with a better OS (2-year OS: 77.8% *vs.* 50.6%, HR = 0.25, *P* = 0.045) and PFS (not reached *vs.* 3 months, HR = 0.13, *P* < 0.001) (27). Furthermore, the author found that there were more CD4^+^ and CD8^+^ T-cells present in the tumor tissue of these patients with early disappearance of circulating T-cells (27). Consistently, in a study of advanced NSCLC treated with ICIs, fewer pretreatment peripheral blood CD8^+^ T-cells were also associated with durable clinical benefit (accuracy = 70%) (28). In addition, the CEA level was also inversely associated with CD8^+^ T-cell count in the TME (29). In our study, CEA was not only significantly inversely associated with MPR but was also significantly inversely associated with T-cell and NK-cell counts in peripheral blood before NCIO.

Comorbidities significantly increase the mortality risk of NSCLC patients (30). Nonetheless, patients with comorbidities were excluded in most trials (31), so understanding of the impact of comorbidities on immunotherapy was poor. In a study of stages III–IV NSCLC, patients were treated with ICIs (*n* = 66), and the DCR and PFS were both superior in the Charlson comorbidity index (CCI) <1 group than in the CCI ≥1 group (94.7% *vs.* 64.3%, *P* < 0.001; 271.0 days *vs.* 232.0 days, *P* = 0.0084) (32). As expected, we found that comorbidities significantly decreased the pCR rate (adjusted OR = 0.16) independently. In an exploratory analysis, comorbidities group had a trend with higher circulating B-cell count after NCIO (median 127.50/µl *vs.* 92.00/µl, *P* = 0.072) (**Complementary Table 7**). An increasing number of studies suggest that B cells have a more critical role than simple bystanders in tumor immunity (33). It is worth noting that studies assessing the effects of B and T-cells were largely concordant (33). A study of neoadjuvant chemotherapy in breast cancer showed that circulating B cells were significantly decreased by > 90% at surgery compared with baseline levels (34). However, NK cells were reduced by nearly 50%, and T-cell counts were lower by 40% (34).

Another interesting finding of our study is that lower TC and HDL-C levels of pre-NCIO significantly correlated with MPR, and TC of pre-NCIO significantly correlated with circulating B-cell rate and NK-cell count of pre-NCIO. To the best of our knowledge, this is the first report of lipid metabolism in NCIO of NSCLC. Emerging evidence has proven that lipid metabolism reprogramming plays important roles in the initiation, development and immunity of tumors (35). Lower TC increased the risk of lung cancer, hepatocellular carcinoma and renal cell carcinoma (36, 37). Cholesterol is a key component in cell membranes, T-cell receptor (TCR) structure, and synaptic function of immune cells (38). Thus, a lower TC may decrease the stability of immune cell membranes as well as cellular homeostasis, especially impacting CD8^+^ T-cell growth and antitumor function (39). However, this may be complicated because increased TC also increases the risk of breast, colorectal, prostate and other cancers (35), and tumor-derived cholesterol correlates with immunosuppression (35). Compared with elevated TC, lower HDL-C was consistently associated with an increased risk of death in patients with most cancers. A meta-analysis showed that a lower HDL-C increased death risk by 37% and relapse risk by 35% across cancers (37). The possible mechanism may be related to HDL-C having anti-oxidation and anti-inflammatory functions (40). However, we found that TC was significantly correlated with the B-cell rate (*r* = 0.404, *P* = 0.033) and NK-cell count (*r* = 0.396, *P* = 0.041), and HDL-C had a trend of correlation with higher CD8^+^ T-cell rate (*r* = 0.314, *P* = 0.070) approached statistical significance. Our findings may have important implications for studies of lipid metabolism in cancer.

The present study had some limitations. First, we did not analyze the relationship between the tumor mutational burden/phenotype of immune cells and the pathological response due to insufficient data. Second, we did not detect the numbers of existing immune cell in the TME. Third, the PFS and OS were immature. Fourth, we cannot exclude some SCC patients with driver oncogene mutations because gene mutation tests are not routine for SCC patients. Fifth, circulating immune cells and SUVmax of tumor were not included in multivariate analyses due to a limited number of cases.

In summary, our study showed that NCIO resulted in a high pCR and MPR rate in stage IIB-IIIC NSCLC. Many factors impacted the incidence of pCR and MPR, five of which were independent factors. Some novel factors, such as peripheral blood CD4^+^ T-cell count/rate, TC and HDL-C of pre-NCIO, are worth further investigation. A clinical predictive model could help to screen a suitable population for NCIO treatment.

## Data availability statement

The raw data supporting the conclusions of this article will be made available by the authors, without undue reservation.

## Ethics statement

The studies involving human participants were reviewed and approved by The Second Xiangya Hospital of Central South University Ethics Committee. Written informed consent for participation was not required for this study in accordance with the national legislation and the institutional requirements.

## Author contributions

XH contributed to data collection, data analyses and article writing. CH, FM and DL contributed to the data analyses. JX, DF, XF and MD contributed to data collection and patient follow-up. PZ and CT contributed to the statistical analyses. YG contributed to language editing and corrections. CL and XL contributed to the conception and design of this study, and CL coordinated the work and reviewed this article. All authors contributed to the article and approved it for publication.
